# Complete locoregional response to radiotherapy and pembrolizumab in an elderly and frail patient with oropharyngeal squamous cell carcinoma: A case report

**DOI:** 10.1097/MD.0000000000047050

**Published:** 2026-03-13

**Authors:** Flaminia Benedetta Zoboli, Mirta Mosca, Ambrogio Gagliano, Viola Laghi, Giambattista Siepe, Karim Rihawi, Elisabetta Nobili, Daria Maria Filippini

**Affiliations:** a Division of Oncology, IRCCS Azienda Ospedaliero-Universitaria di Bologna, Bologna, Italy; b Radiation Oncology, IRCCS Azienda Ospedaliero-Universitaria di Bologna, Bologna, Italy; c Department of Medical and Surgical Sciences (DIMEC), Alma Mater Studiorum, Università di Bologna, Bologna, Italy.

**Keywords:** advanced disease, case report, head and neck squamous cell carcinoma, immunotherapy, radiotherapy

## Abstract

**Rationale:**

Immune checkpoint inhibitors (ICIs) have revolutionized the management of head and neck squamous cell carcinoma, particularly in the recurrent/metastatic setting. Although the combination of ICIs and radiotherapy (RT) in locally advanced disease has not yet demonstrated clear survival benefits, the strong biological rationale for their synergistic action continues to support investigation, especially in frail or elderly patients unfit for standard chemoradiotherapy.

**Patient concerns:**

An 83-year-old male presented with odynophagia, dysphagia, significant weight loss, and right cervical swelling, all impairing oral intake and quality of life.

**Diagnoses:**

Moderately differentiated squamous cell carcinoma (G2) of the oropharynx (right base of tongue), human papillomavirus-negative, programmed death-ligand 1 combined positive score = 30. The tumor was staged as cT4a cN3b M1.

**Interventions:**

The patient underwent hypofractionated RT (50 Gy in 20 fractions) delivering only to the primary lesion, followed by pembrolizumab 200 mg every 3 weeks. One pulmonary oligoprogressive lesion was treated with stereotactic body RT (60 Gy in 8 fractions). After 29 cycles of pembrolizumab, treatment was discontinued due to the onset of immune-related grade 3 hepatotoxicity. Two additional pulmonary oligoprogressive lesions were treated with stereotactic body radiotherapy, 55 Gy in 5 fractions.

**Outcomes:**

A complete response was achieved and maintained at both the primary tumor and nodal sites. Following immunotherapy discontinuation, liver biopsy confirmed immune-related cholangitis. Despite persistent elevation of cholestatic markers, disease control on T and N was preserved. A new pulmonary oligoprogression is currently under active surveillance.

**Lessons:**

This case supports the hypothesis of synergism between RT and immunotherapy in an elderly and frail patient with advanced head and neck squamous cell carcinoma. Hypofractionated RT on the primary tumor alone, combined with ICIs, may lead to sustained locoregional control. Immune-related hepatic toxicity, while clinically significant, did not preclude stable disease.

## 1. Introduction

In recent years, immune checkpoint inhibitors (ICIs) have revolutionized the treatment landscape of different tumor types, including head and neck squamous cell carcinoma (HNSCC) in the recurrent/metastatic (R/M) setting.^[1]^ The landscape of immunotherapy in R/M HNSCC has been rapidly evolving, with numerous clinical studies published over the last few years. While landmark trials leading to regulatory approvals of ICIs are well-known within the medical community, several other studies have been negative, failing to demonstrate a survival benefit for HNSCC patients.^[2]^

ICIs have also been tested in the setting of locally advanced, potentially curable disease in association with radiotherapy (RT), showing good tolerability but no clear advantage in clinical outcomes to date. Nevertheless, immune-RT combinations may enhance the antitumoral immune response: local radiation can trigger immunogenic cell death, promoting systemic inflammation and immune activation through dendritic cell priming and cytotoxic T cell recruitment, ultimately fostering anticancer immunity.^[3]^

Here, we report the case of an elderly and frail patient with human papillomavirus (HPV)-negative oropharyngeal squamous cell carcinoma (OPSCC) who achieved a complete locoregional response with hypofractionated RT delivered exclusively to the primary tumor, in combination with pembrolizumab. Neck lymph node metastases (N) regressed without direct irradiation, supporting the hypothesis of a systemic immunologic effect induced by RT. The patient initially experienced clinical benefit and a stability of lung metastasis treated with stereotactic body radiotherapy (SBRT). After 29 cycles of pembrolizumab, treatment was discontinued due to immune-related hepatotoxicity, histologically confirmed as chronic hepatitis with acute cholangitis. Despite this adverse event, the patient maintained complete response on the primary tumor (T) and N. The oligoprogressive lung disease was managed with SBRT.

This case highlights the potential of immunotherapy as a single agent associated with RT directed to the primary tumor and oligoprogressive lesions, to achieve durable disease control in frail patients with advanced, HPV-negative OPSCC. It also emphasizes the critical role of vigilant and multidisciplinary management of immune-related toxicities.

## 2. Case report

An 83-year-old male patient, active smoker (60 pack/years), with a history of urothelial papillary carcinoma G2 pTa surgically treated in May 2024, chronic obstructive pulmonary disease, peripheral arterial disease, previous cerebral hemorrhage, dyslipidemia, and hypertension, was diagnosed with OPSCC in July 2021. At the diagnosis, his Eastern Cooperative Oncology Group Performance Status was 2 due to disease-related pain and malnutrition.

Because of odynophagia and dysphagia, both limiting the oral nutritional intake with a subsequent weight loss, he underwent an ear, nose, and throat examination, which showed a partially ulcerated vegetative lesion of the right tongue base, and a 3-cm right omolateral cervical adenopathy localized at the II neck level.

Histological report showed an infiltrating squamous cell carcinoma, G2; p16 and HPV-DNA were both negative. A staging computed tomography (CT) scan was then performed, detecting lung bilateral metastases.

**Figure 1.**
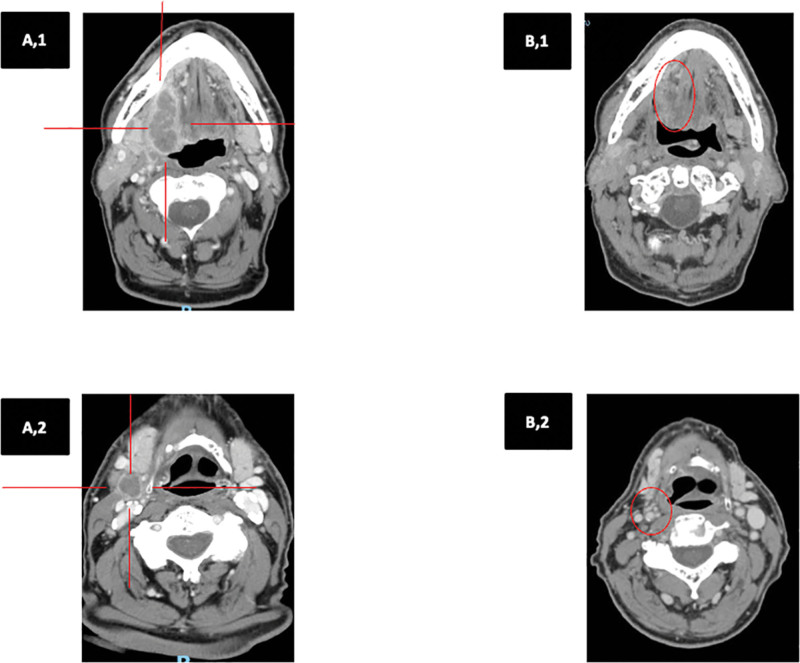
Computed tomography (CT) scan showing the presence of the primary lesion on the base of tongue (A,1) and omolateral lymph node metastases (A,2) at initial diagnosis and the complete local (B,1) and regional (B,2) response after treatment with radiotherapy and 20 cycles of pembrolizumab.

The disease was staged as cT4a (due to the engagement of extrinsic tongue muscles), cN3b (extranodal extension) M1, IVC (according to American Joint Committee on Cancer, eighth edition). Fig. 1 (A,1 and A,2) shows the presence of locoregional disease at initial diagnosis.

### 2.1. The programmed death-ligand 1 combined positive score value was 30

In August 2021, after multidisciplinary discussion and based on tumor burden and both the patient’s comorbidities and symptoms (dysphagia and severe uncontrolled pain), an antalgic and cytoreductive hypofractionated RT was performed on the primary tumor lesion localized at the right tongue base. From August to September 2021, the patient received a total dose of 50 Gy in 20 fractions (dose per fraction: 2.5 Gy). After 5 days into treatment, he was started on a first-line immunotherapy with pembrolizumab 200 mg flat-dose every 3 weeks.

After receiving RT and 2 concomitant cycles of pembrolizumab, the patient experienced a significant clinical benefit, which allowed him to resume oral nutrition and to decrease opioid dosage as a result of improved pain control. Upon completion of RT, the patient continued immunotherapy with pembrolizumab every 3 weeks.

In December 2021, after 7 cycles of ICI, an ^18^F-fluoro-2-deoxy-D-glucose-positron emission tomography documented a metabolic normalization of the primary lesion as well as of the lymphnodal metastases while showing a metabolic increase of 1 lung metastasis at the upper right lobe.

Based on the locoregional complete response achieved, the good treatment tolerance, and the clinical benefit, pembrolizumab was continued, but in May 2022, SBRT was performed on the increasing right lung metastasis (60 Gy in 8 fractions), obtaining a reduction of the lung lesion. Subsequent tumor reassessments confirmed the locoregional complete response with a reduction of the irradiated lung lesion and stability of the other lung lesions.

The patient continued pembrolizumab 200 mg flat-dose every 3 weeks, receiving a total of 29 cycles; he maintained a locoregional complete response (Fig. 1B,1 and B,2) and a response of lung metastasis.

In April 2023, routine blood tests revealed progressive elevation in transaminases (peak: aspartate aminotransferase/alanine aminotransferase 146/162 U/L, GGT 792 U/L). The patient developed pruritus, without jaundice, fever, or signs of chronic liver disease. Imaging (both ecography and CT scan) excluded biliary obstruction or hepatic metastases; blood tests excluded viral, autoimmune, and metabolic causes. An initial therapeutic attempt with budesonide and ursodeoxycholic acid did not prevent worsening of liver enzymes. Despite negative autoantibody profiles, a mixed hepatocellular-cholestatic damage due to pembrolizumab remained the most plausible diagnosis. Notably, anti-hepatitis B core antibodies were detected, suggesting that past hepatitis B virus contact (hepatitis B virus-DNA was negative), a very low but non-negligible risk of reactivation exists under immunosuppression.

Tauro-ursodeoxycholic acid and budesonide stabilized cholestasis markers but did not induce significant improvement. As the liver showed no signs of cirrhosis, systemic steroid therapy at 0.5 mg/kg was proposed as prophylaxis prior to any rechallenge with immunotherapy, without any benefit.

In September 2023, hepatic elastography revealed moderately elevated stiffness values, and a percutaneous liver biopsy confirmed the suspicion of immune-related hepatitis, showing features of chronic hepatitis with moderate activity and acute cholangitis. After multidisciplinary evaluation with hepatologists, this hepatic profile was considered a negative prognostic factor for restarting immunotherapy, especially in light of complete locoregional oncologic response, and second-line immunosuppressive agents were considered inappropriate due to patient age, comorbidities, and risk of severe adverse events. The patient continued with tauro-ursodeoxycholic acid and budesonide therapy, and serial blood tests were performed, showing progressive improvement of hepatic values without the need for further immunosuppression.

In December 2022, radiologic exams (both CT scans and FDG-positron emission tomography) documented a persistent complete response of the primary lesion as well as of the lymphnodal metastases while showing a slow metabolic and dimensional increase of 2 lung metastases at the inferior right lobe and medium lobe. After multidisciplinary discussion and based on the locoregional complete response achieved and the clinical benefit, SBRT was performed on the increasing inferior right lobe lung metastasis in January 2024 (55 Gy in 5 fractions) and on the medium lobe lung metastasis in July 2024 (27 Gy in 1 fraction), obtaining a reduction of the lung lesions.

Until today (46 months from the diagnosis), the patient, Eastern Cooperative Oncology Group Performance Status 1, maintains a locoregional complete response and a slow progressive disease on lung metastases under close surveillance. His quality of life (QoL) has remained satisfactory.

## 3. Discussion

Several data suggest that RT could modify tumor microenvironment in a pro-inflammatory way, promoting inflammatory cell death, upregulation of major histocompatibility complex I and leading to the release of neo-antigens by tumor cells. Radiation can also induce the release of cytosolic DNA, which stimulates the cyclic GMP-AMP synthase/stimulator of interferon genes pathway, ultimately leading to the upregulation of interferon gamma production and the subsequent activation of dendritic cells.^[3]^

In this context, it is important to identify the proper timing to combine immunotherapy and RT. When administered before RT, ICIs may increase radiation efficacy by creating an active immune microenvironment, whereas when RT is given concomitantly with immunotherapy, ICIs may find more tumor-specific antigens generated by RT. However, concomitant delivery may increase the risk of toxicity. Hypofractionated RT schemes may mitigate this by limiting lymphocyte depletion and reducing both acute and late toxicities because it causes lower damage to circulating blood, sparing circulating lymphocytes, than a conventional RT schedule.

However, beyond the setting of locally advanced disease, important evidence has emerged regarding the role of SBRT in oligometastatic cancers. In the SABR-COMET phase II randomized trial by Palma et al which included a broad spectrum of histologies, SBRT significantly improved overall survival (OS) with long-term durable benefit, without impacting QoL during extended follow-up.^[4]^ Similarly, in the more recent GORTEC 2014-04 “OMET” randomized phase II trial, Thariat et al demonstrated that in patients with oligometastatic head and neck cancers, SBRT alone achieved survival outcomes comparable to SBRT combined with EXTREME-regimen chemotherapy, but with less deterioration in QoL and lower toxicity rates.^[5]^

Specific to metastatic HNSCC, the only randomized phase II trial available, conducted by McBride et al compared nivolumab alone versus nivolumab combined with SBRT. Although the trial reported no significant improvements in objective response rate, progression-free survival (PFS), or OS, the combination was well-tolerated, highlighting the safety of SBRT in association with ICIs in this population.^[6]^

Some recent trials studying the combination of immunotherapy and RT in locally advanced HNSCC have shown a good safety profile, but no significant improvement in clinical outcomes.

A recent single-center retrospective study by Chen et al (2025)^[7]^ evaluated the safety and efficacy of re-irradiation combined with pembrolizumab in patients with locally recurrent HNSCC. While their study population consisted of patients undergoing re-irradiation for locoregionally recurrent disease, our case represents a de novo metastatic scenario treated with first-line immunoradiotherapy. In contrast to Chen’s study, where RT was directed to both primary and nodal sites, our patient received irradiation only on the primary tumor with regression of unirradiated nodal metastases, supporting the hypothesis of a systemic immune-mediated effect. Despite different clinical settings, both findings converge on the potential synergy between RT and ICIs in HNSCC, warranting further investigation in prospective trials.

The PembroRad phase II trial enrolled cisplatin-ineligible patients with locally advanced HNSCC, randomizing them to receive RT with either cetuximab or pembrolizumab. Pembrolizumab was well-tolerated but was not superior to the cetuximab-based arm in terms of locoregional control at 15 months from the end of RT.^[8]^ Similarly, the phase III placebo-controlled JAVELIN head and neck 100 trial randomized 697 patients to receive standard chemo-RT (3-weekly cisplatin with 70 Gy of intensity-modulated RT, 2 Gy per fraction) with or without concurrent and adjuvant avelumab. Although avelumab was well-tolerated, it failed to improve progression-free survival or OS compared with conventional chemo-RT.^[9]^ Comparable negative results were obtained with pembrolizumab in the recent phase III KEYNOTE-412 trial.^[10]^

Currently, multiple clinical trials are ongoing (NCT03386357, NCT04862455, NCT03283605, NCT05136768, NCT03070366), exploring different combinations and timings of immunotherapy with RT in metastatic HNSCC. The hypothesis behind multisite irradiation combined with ICIs is to enhance therapeutic synergy by simultaneously reducing overall tumor burden, stimulating a broader and more potent radiation-induced immune response from a diverse T cell repertoire, and maintaining local control over lesions that might otherwise cause significant morbidity. Future research is critically needed to better define the optimal radiation parameters— – specifically dose, fractionation, and sequencing – in order to maximize the synergistic potential of immunoradiotherapy strategies in head and neck cancer.

One of the speculations about the failure of ICIs and RT combination in locally advanced settings could be that the irradiation on lymph node metastases could countermeasure the positive effect of ICIs.^[11]^ One emerging hypothesis regarding the failure of some ICI + RT combinations in locally advanced settings is that the irradiation of regional lymph node metastases may impair immune priming and reduce the therapeutic efficacy of immunotherapy.^[11]^ Various preclinical and translational studies have shown that tumor-draining lymph nodes are crucial sites for early T cell activation following ICI administration.^[12,13]^ These lymph nodes host a specialized microenvironment where dendritic cells and type I interferons (IFN-I) orchestrate the recruitment and activation of naïve and memory CD8^+^ T cells. In fact, upregulation of CD8^+^ T cells and activated CD8^+^ subsets has been demonstrated in tumor-draining lymph nodes, but not in nondraining or tumor-free nodes, highlighting their specific role in mediating effective antitumor responses after PD-1/programmed death-ligand 1 blockade. These findings strongly support the rationale for lymphatic-preserving RT approaches when combined with ICIs, particularly in the immediate period following immune checkpoint inhibition. Elective nodal irradiation, by contrast, may damage these functional immune niches, suppress dendritic cell activity, and reduce T cell diversity, thus limiting the systemic and regional impact of immunotherapy.

Interestingly, in our case, the RT was only performed on the primary tumor, leading to a complete local and regional response. This observation supports the idea that preserving the immune competence of lymphatic tissues – particularly in frail or immunologically vulnerable patients – may enhance responsiveness to ICIs. However, studies confirming such a hypothesis are warranted. In the R/M setting, evidence regarding the efficacy of ICIs and concomitant RT is lacking. In this setting, radiation could stimulate a systemic antitumor effect, producing the abscopal effect (tumor regression in a site distant from the localized irradiation), which has been observed in melanoma and non-small cell lung cancer patients treated with ipilimumab and concomitant RT.^[11]^

Based on a strong biological rationale, several clinical trials evaluating combinations of ICIs and RT in R/M HNSCC are still ongoing.

This case report is limited by its nature as a single-patient observation, and thus, its findings cannot be generalized. Therefore, prospective studies with larger cohorts are necessary to validate the potential synergistic effect of hypofractionated RT and ICIs in this population. Additionally, although the patient-reported improved symptoms and maintenance of a satisfactory QoL, no standardized patient-reported outcome measures were used to objectively assess this aspect.

As we await further data, studies testing the most beneficial timing combination of ICIs following radiation therapy with a hypofractionated scheme could be considered for disease control in selected patients.

## Author contributions

**Conceptualization:** Daria Maria Filippini.

**Data curation:** Flaminia Benedetta Zoboli, Mirta Mosca, Daria Maria Filippini.

**Funding acquisition:** Daria Maria Filippini.

**Investigation:** Ambrogio Gagliano, Daria Maria Filippini.

**Methodology:** Flaminia Benedetta Zoboli, Viola Laghi, Karim Rihawi, Elisabetta Nobili, Daria Maria Filippini.

**Project administration:** Daria Maria Filippini.

**Resources:** Giambattista Siepe, Daria Maria Filippini.

**Supervision:** Daria Maria Filippini.

**Writing – original draft:** Flaminia Benedetta Zoboli.

**Writing – review & editing:** Flaminia Benedetta Zoboli, Daria Maria Filippini.
